# Progesterone and Allopregnanolone Rapidly Attenuate Estrogen-Associated Mechanical Allodynia in Rats with Persistent Temporomandibular Joint Inflammation

**DOI:** 10.3389/fnint.2020.00026

**Published:** 2020-05-08

**Authors:** Rebecca S. Hornung, William L. Benton, Sirima Tongkhuya, Lynda Uphouse, Phillip R. Kramer, Dayna Loyd Averitt

**Affiliations:** ^1^Department of Biology, Texas Woman’s University, Denton, TX, United States; ^2^Department of Biomedical Sciences, Texas A&M University College of Dentistry, Dallas, TX, United States

**Keywords:** orofacial pain, progesterone, estrogen, mechanical allodynia, temporomandibular joint, inflammatory pain, allopregnanolone, gonadal hormones

## Abstract

Temporomandibular joint disorder (TMD) is associated with pain in the joint (temporomandibular joint, TMJ) and muscles involved in mastication. TMD pain dissipates following menopause but returns in some women undergoing estrogen replacement therapy. Progesterone has both anti-inflammatory and antinociceptive properties, while estrogen’s effects on nociception are variable and highly dependent on both natural hormone fluctuations and estrogen dosage during pharmacological treatments, with high doses increasing pain. Allopregnanolone, a progesterone metabolite and positive allosteric modulator of the GABA_A_ receptor, also has antinociceptive properties. While progesterone and allopregnanolone are antinociceptive, their effect on estrogen-exacerbated TMD pain has not been determined. We hypothesized that removing the source of endogenous ovarian hormones would reduce inflammatory allodynia in the TMJ of rats and both progesterone and allopregnanolone would attenuate the estrogen-provoked return of allodynia. Baseline mechanical sensitivity was measured in female Sprague–Dawley rats (150–175 g) using the von Frey filament method followed by a unilateral injection of complete Freund’s adjuvant (CFA) into the TMJ. Mechanical allodynia was confirmed 24 h later; then rats were ovariectomized or received sham surgery. Two weeks later, allodynia was reassessed and rats received one of the following subcutaneous hormone treatments over 5 days: a daily pharmacological dose of estradiol benzoate (E2; 50 μg/kg), daily E2 and pharmacological to sub-physiological doses of progesterone (P4; 16 mg/kg, 16 μg/kg, or 16 ng/kg), E2 daily and interrupted P4 given every other day, daily P4, or daily vehicle control. A separate group of animals received allopregnanolone (0.16 mg/kg) instead of P4. Allodynia was reassessed 1 h following injections. Here, we report that CFA-evoked mechanical allodynia was attenuated following ovariectomy and daily high E2 treatment triggered the return of allodynia, which was rapidly attenuated when P4 was also administered either daily or every other day. Allopregnanolone treatment, whether daily or every other day, also attenuated estrogen-exacerbated allodynia within 1 h of treatment, but only on the first treatment day. These data indicate that when gonadal hormone levels have diminished, treatment with a lower dose of progesterone may be effective at rapidly reducing the estrogen-evoked recurrence of inflammatory mechanical allodynia in the TMJ.

## Introduction

Women are genetically predisposed to experiencing certain chronic pain disorders. Some chronic pain conditions, such as endometriosis (Mehedintu et al., 2014), vulvodynia (Hoffstetter and Shah, 2015), and menstrual pain, are female-specific. Further, chronic pain disorders that affect both genders but are more prevalent in women include fibromyalgia, irritable bowel syndrome, temporomandibular joint disorders (TMD), Raynaud’s syndrome, rheumatoid arthritis, multiple sclerosis, and migraine (Wei et al., 2018). Further, there is a gender disparity not only in the predominance of pain disorders reported in women but also pain perception. Women report a greater sensitivity to pain (Rosseland and Stubhaug, 2004; Fillingim et al., 2009; Kim et al., 2013), lower pain threshold, and less tolerance to pain (Fillingim et al., 2009; IOM, 2011). This gender disparity in increased pain and/or pain sensitivity may be attributed to ovarian hormones. In support, pain sensitivity or intensity also vary during the menstrual cycle (Marcus, 1995; Unruh, 1996; Riley et al., 1999; Sherman and LeResche, 2006; Craft, 2007; Martin, 2009; Teepker et al., 2010). Exogenous ovarian hormones also affect pain such that oral contraceptives, which result in more constant hormone levels, can improve pain symptoms (Coffee et al., 2007; Craft, 2007; Sulak et al., 2007). However, exogenous hormones also increase pain sensitivity. Transgender individuals who undergo physical transition from male to female are usually prescribed a dose of estrogen equivalent to the recommended dose for hormone replacement therapy (HRT) in postmenopausal women (Moore et al., 2003). These individuals report an increase in pain, whereas, the individuals that transition physically from female to male report a significant improvement in pre-existing pain conditions (Aloisi et al., 2007).

Estrogen can be pronociceptive (Wu et al., 2010; Kou et al., 2011; Zhang et al., 2012a,b; Ralya and McCarson, 2014; Pratap et al., 2015; Bi et al., 2017) and can upregulate inflammatory mediators (Kou et al., 2011; Puri et al., 2011), however, antinociceptive properties of estrogen have also been reported (Fischer et al., 2008; Fávaro-Moreira et al., 2009; Kramer and Bellinger, 2013). Together, the literature indicates that pharmacological doses of estrogen tend to increase pain. Progesterone and its metabolite, allopregnanolone, on the other hand, have well-documented anti-inflammatory (He et al., 2004; VanLandingham et al., 2007; Labombarda et al., 2011; Garay et al., 2012; Coronel et al., 2014, 2016b; Grandi et al., 2016) and antinociceptive effects on neuropathic pain (Charlet et al., 2008; Coronel et al., 2011; Kawano et al., 2011; Afrazi and Esmaeili-Mahani, 2014; Jarahi et al., 2014; Liu et al., 2014; Huang et al., 2016). Progesterone and allopregnanolone have been reported to reduce neuropathic pain in animal models of chemotherapy-induced neuropathy (Meyer et al., 2010, 2011), sciatic nerve crush or constriction (Roglio et al., 2008; Coronel et al., 2011; Huang et al., 2016), diabetic-neuropathy (Leonelli et al., 2007; Afrazi and Esmaeili-Mahani, 2014), and trigeminal nerve root demyelination (Kim et al., 2012). These studies used a pharmacological dose of progesterone (e.g., 16 mg/kg) in their treatments.

One major pain disorder that is more prevalent in women is temporomandibular joint disorder (TMD; Liu and Steinkeler, 2013). TMD affects approximately 10% of the population (LeResche, 1997) and is more prevalent in women (Unruh, 1996; Berkley, 1997; Isong et al., 2008; Manson, 2010; Bartley and Fillingim, 2013), which account for 75% of all cases reported (Macfarlane et al., 2001). While the causes of TMD are well established, the underlying mechanisms that make TMD pain more prevalent and severe in women is unclear, but the ovarian hormones estrogen and progesterone have been implicated. TMD pain intensifies at the onset of puberty, is highest during child-bearing years, and dissipates during pregnancy and after menopause (LeResche et al., 2003). TMD pain has been reported to reemerge in some post-menopausal women undergoing HRT, particularly, pharmacological estrogen replacement therapy (LeResche et al., 1997; Wise et al., 2000).

Estrogen, when administered in a pharmacological dose, appears to aggravate TMD pain. In animal models, high estrogen enhances temporomandibular joint (TMJ) nociception (Cairns et al., 2002; Bereiter et al., 2005; Flake et al., 2005; Okamoto et al., 2008; Kou et al., 2011, 2014; Bi et al., 2017), although the opposite has also been reported (Fischer et al., 2008). High estrogen upregulates pro-inflammatory cytokines in the TMJ (Yun et al., 2008; Kou et al., 2011, 2014; Xue et al., 2018) and voltage-gated sodium channels in the trigeminal ganglia resulting in hyperalgesia (Bi et al., 2017). On the other hand, physiological levels can upregulate GABA_A_ receptor subunit expression in the trigeminal ganglia (Puri et al., 2011) resulting in a decrease in nociceptive behavior (Kramer and Bellinger, 2013, 2014). Estrogen treatment decreases action potential thresholds in TMJ afferents (Flake et al., 2005) and increases reflex jaw muscle activity which was reduced by ovariectomy (Cairns et al., 2002). At second order neurons, estrogen increases neural activity and excitability within the trigeminal nucleus caudalis of the medullary spinal cord (Bereiter et al., 2005; Okamoto et al., 2008).

While estrogen seems to have a complicated role, the contribution of progesterone has been largely overlooked in research on TMD. It has been reported that both estrogen and progesterone decrease formalin and glutamate-evoked TMJ nociceptive behaviors and progesterone reduces pro-inflammatory cytokines in the TMJ (Xue et al., 2017). Of the studies reporting the effects of estrogen and progesterone on TMD, no studies have examined the effects of ovariectomy on established mechanical allodynia at the inflamed TMJ and then re-examined sensory thresholds following the re-introduction of a pharmacological dose of estrogen and pharmacological to sub-physiological doses of progesterone. Therefore, we hypothesized that removal of endogenous ovarian hormones would attenuate inflammatory allodynia at the TMJ, which would return following high estrogen treatment. We further hypothesized that progesterone and its metabolite allopregnanolone would attenuate the return of inflammatory orofacial mechanical allodynia in female rats. These hypotheses were tested in the present experiments designed to determine if: (1) the removal of endogenous ovarian hormones would attenuate TMD allodynia, modeling clinical reports of reduced TMD pain in post-menopausal women; (2) whether the reintroduction of exogenous estrogen would trigger the return of TMD allodynia; and (3) whether exogenous progesterone or its major metabolite could dampen reemergence of TMD allodynia.

## Materials and Methods

### Subjects

A total of 97 adult Sprague–Dawley female rats (150–175 grams; Charles River Laboratories, Wilmington, MA, USA) were used in these experiments. Rats were housed two per cage in a colony room with a 12:12 h light:dark cycle (lights on at 8 a.m.). Food and water were available *ad libitum*. Rats were allowed 1 week to acclimate to the facility before experiments began. Vaginal lavages were conducted between 9 a.m. and 11 a.m. for 10 days or two consecutive cycles to ensure rats were cycling properly. Estrus was determined by the predominance of cornified epithelial tissue and proestrus was determined by the predominance of nucleated epithelial tissue. Diestrus I was differentiated from diestrus II by the presence of leukocytes. All studies were approved by Texas Woman’s University Institutional Animal Care and Use Committee. Experiments conformed to federal guidelines and the committee for Research and Ethical Issues of the International Association for the Study of Pain.

### Drugs

Stock solutions of estradiol benzoate (E2), progesterone (P4), and 5α-Pregnan-3α-ol-20-one (allopregnanolone; Sigma Aldrich, St. Louis, MO, USA) were dissolved in sesame seed oil (Sigma Aldrich, St. Louis, MO, USA), diluted prior to injection, and administered subcutaneously (s.c).

### Ovariectomy

Female rats were deeply anesthetized (induction 3%; maintenance 2.5%) with inhalation of gas (isoflurane, USP, Henry Schein Animal Health, Dublin, OH, USA) anesthesia. Topical lidocaine was applied before a single incision was made to the anterolateral abdominal area. The abdominal muscle was cut, ovaries were ligated with 3-0 Vicryl sutures, and excised. Abdominal muscle was sutured with 3-0 Vicryl sutures and the epidermal layer was stapled with an Autoclip Wound Closing System (Braintree Scientific, Braintree, MA, USA). Immediately before and 24 h following surgery, animals were administered the antibiotic Baytril (0.02 ml of a 22.7 mg/kg solution leading to approximately 4.4 mg/kg dose) intramuscularly (i.m.). The analgesic Rimadyl (0.03 ml of a 50 mg/kg solution leading to approximately 2.5 mg/kg dose) was administered subcutaneously (s.c.) immediately following surgery. Rats that received sham surgery received the same procedural manipulations and treatments except for removal of ovaries. Rats were allowed 2 weeks recovery from surgery and for the elimination of endogenous ovarian hormones.

### Temporomandibular Joint Inflammation

Complete Freund’s adjuvant (CFA; 30 μl 1 mg/ml; CFA; mycobacterium tuberculosis; Sigma–Aldrich) was dissolved 1:1 in saline solution and injected under brief isoflurane gas anesthesia into the intra-articular area of the TMJ. The TMJ area was palpated for the TMJ, confirmed by movement of the mandible, then the needle was directed to the joint and injected with CFA using a 30-gauge needle.

### Behavior Testing

Behavior was tested before CFA injections, 24 h after CFA injections, then 2 weeks after OVX. Von Frey filaments (North Coast Medical Inc., Gilroy, CA, USA) were utilized to test the force to withdrawal threshold as a measure of mechanical allodynia at the cutaneous tissues surrounding the inflamed TMJ, as previously reported (Ren, 1999). For this test, a starting filament was first applied to the TMJ; 2.0-g for non-inflamed tissues and 0.16-g filament for inflamed tissue (Ren, 1999; Villa et al., 2010). If no response was observed, 30 s later the next thickest filament was applied, and so on until a withdrawal response was observed. The thickest filament that elicited a withdrawal response was 10.0 g; however, most animals withdrew their head to the 6.0 g filament. If a withdrawal response was observed with the starting filament, 30 s later the next thinnest filament was applied, and so on until no withdrawal response was observed. The filament size that produced at least three responses was recorded as the threshold grams of pressure required to elicit a withdrawal response as a measure of mechanical allodynia.

### Hormone Treatments

To test the role of P4 after 5 days of hormone treatment, animals received one of the following hormone treatments s.c. every day for 5 days: (a) daily pharmacological dose of estradiol benzoate (E2; 50 μg/kg; Fischer et al., 2008); (b) daily pharmacological dose of progesterone (P4, 16 mg/kg); (c) daily E2 and P4 (16 mg/kg); (d) daily E2 and intermittent progesterone; or (e) vehicle (sesame seed oil) control and behavior testing was completed 1 h after last hormone injection. To determine if P4’s effects on mechanical allodynia occurred prior to the 5th day of hormone treatments, animals received one of the following hormone treatments s.c. every day for 5 days: (a) daily pharmacological dose of E2 (50 μg/kg); (b) daily pharmacological dose of P4 (16 mg/kg); (c) daily E2 and P4 (16 mg/kg); (d) daily E2 and intermittent progesterone; or (e) vehicle control and behavior testing was completed 1 h after hormone injections. To determine if physiological to sub-physiological doses of P4 would be effective in attenuating the return of mechanical allodynia, animals received one of the following hormone treatments s.c. every day for 5 days: (a) daily pharmacological dose of E2 (50 μg/kg); (b) daily P4 (16 μg/kg or 16 ng/kg); (c) daily E2 and P4 (16 μg/kg or 16 ng/kg); (d) daily E2 and intermittent progesterone; or (e) vehicle control and behavior testing was completed 1 h after hormone injections. To test the effects of allopregnanolone, a separate group of rats received one of the following hormone treatments: (a) daily E2 (50 μg/kg); (b) daily allopregnanolone (0.16 mg/kg); (c) daily E2 and continuous allopregnanolone; (d) daily E2 and intermittent allopregnanolone; or (e) vehicle control and behavior testing was completed 1 h after hormone injections. The pharmacological dose of progesterone (16 mg/kg) was chosen from previous studies reporting attenuation of mechanical allodynia in a rat model of spinal cord injury-induced neuropathic pain (Coronel et al., 2011, 2014). The two lower doses (16 μg/kg or 16 ng/kg) of progesterone were chosen to include physiological to sub-physiological doses. The dose of allopregnanolone was chosen from a previous study reporting attenuation of mechanical allodynia in a rat model of post-operative neuropathic pain (Fujita et al., 2018).

### Progesterone Radioimmunoassay

A separate group of 20 female rats were used to measure serum progesterone levels. Rats received CFA injections into the intra-articular area of the TMJ and were ovariectomized 24 h later. Two weeks post-OVX, animals received one of the following hormone treatments: (a) E2 (50 μg/kg); (b) E2 and P4 (16 mg/kg); (c) E2 and P4 (16 μg/kg); (d) E2 and P4 (16 ng/kg; *n* = 5 per group). One hour after hormone treatment, animals were rapidly decapitated under gas anesthesia (isoflurane; 3%) and trunk blood was collected in BD vacutainer^®^ spray-coated K2EDTA collection tubes (Pulmolab, CA, USA) on ice. Immediately after collection, blood was centrifuged at 3,000 rpm for 15 min. Serum was separated and stored at −20°C. After diethyl ether extraction, P4 levels were measured with a Progesterone Radioimmunoassay Kit (Immuno-Biological Laboratories Inc.; Minneapolis, MN, USA) according to the manufacturer’s instructions.

### Data Analysis

Behavioral data were presented as the mean ± standard error of the mean of the force in grams (g) required to elicit a withdraw as a measure of the degree of mechanical allodynia. Data were analyzed by one-way analysis of variance (ANOVA) and two-way repeated measures ANOVA using GraphPad Prism 7 with time as the repeated measure and treatment as the independent factor. Tukey’s *post hoc* analysis was conducted. Statistical significance was tested at *p* ≤ 0.05. Radioimmunoassay data were presented as the mean ± standard error of the mean (range) in ng/ml and analyzed by Mann–Whitney *t*-test and statistical significance was tested at *p* ≤ 0.05.

## Results

### Ovariectomy Reverses CFA-Evoked Mechanical Allodynia in the TMJ

**Figure 1:**
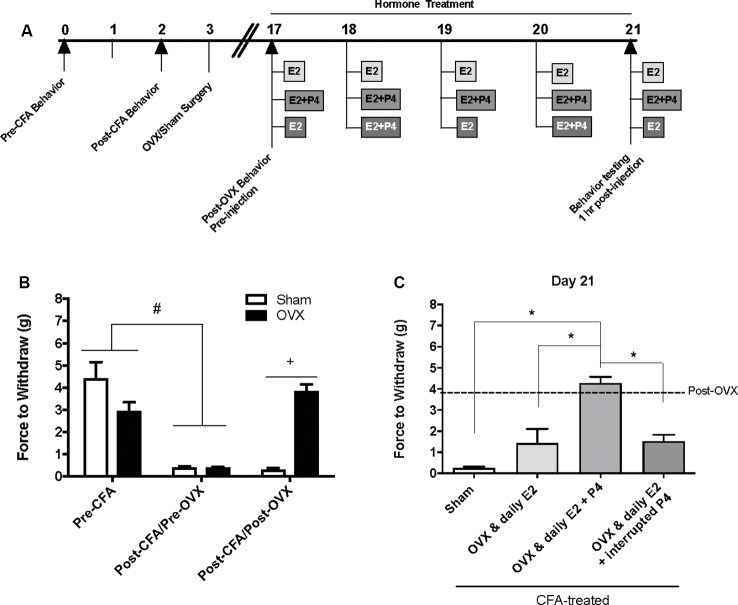
Effects of ovariectomy and gonadal hormone treatment on complete Freund’s adjuvant (CFA)-evoked mechanical allodynia in the inflamed rat temporomandibular joint. **(A)** Experimental timeline of behavior testing, ovariectomy, and hormone treatments administered. Behavior testing was done 24-hrs prior to and following CFA injections followed by either ovariectomy (OVX; *n* = 19) or sham surgery (*n* = 5). Two-weeks later behavior testing occurred followed by hormone treatments. Hormones were administered every day for 5 days except interrupted progesterone, which was administered on days 2 and 4. Behavior testing occurred 1 h after hormone injection on day 5. **(B)** Bar graph showing CFA-evoked mechanical allodynia (pre-CFA vs. post-CFA/pre-OVX) followed by sham (open bars) vs. OVX (closed bars) surgery (post-CFA/post-OVX). **(C)** Effects of hormone treatment 1 h after last treatment on day 21 following a five-day hormone treatment regimen of a daily pharmacological dose of estradiol (50 μg/kg E2; *n* = 5), daily E2 and daily pharmacological dose of progesterone (50 μg/kg E2 + 16 mg/kg P4; *n* = 7), or daily E2 with interrupted P4 (*n* = 5) compared to sham treated with daily vehicle (sesame seed oil, open bars; *n* = 5). ^#^Indicates significant difference between pre-CFA and post-CFA/pre-OVX groups. ^+^Indicates significant difference between OVX and sham groups post-surgery. *Indicates significant difference between hormone treatment groups. Statistical significance was tested at *p* ≤ 0.01.

Baseline mechanical threshold was measured before and after CFA injections then again 2 weeks after OVX or sham surgery (see timeline Figure 1A). There was a significant interaction between the treatment groups across time (*F*_(2,44)_ = 20.6; *p* <0.05). CFA evoked a significant reduction in the force to withdrawal in all rats (*p* < 0.05; Figure 1B). Following ovariectomy, the force required to elicit withdraw (in grams) was significantly greater in ovariectomized rats (closed bars; *p* <0.05) compared to sham rats which remained allodynic (open bars). TMJ inflammation did not cause weight loss as animal weight 2 weeks post-surgery was significantly greater than animal weight prior to CFA injections (*F*_(1.859,117.1)_ = 441.4; *p* <0.05; data not shown).

### E2 (50 μg/kg) Treatment Elicits Return of CFA-Evoked Mechanical Allodynia in the TMJ

Following OVX, rats received 5 days of hormone treatment (see treatment groups Figure 1A) and mechanical allodynia was reassessed 1 h following the last injection day (day 21 on timeline; day 5 of hormone treatment). There was a significant effect of treatment (*F*_(3,18)_ = 22.92; *p* <0.05). Ovariectomized rats that received daily E2 with daily P4 (mid gray bars) retained significantly higher mechanical thresholds (*p* <0.05), while rats that received only daily E2 (light gray bars) displayed similar mechanical allodynia to sham animals that received vehicle injections (open bars; *p* > 0.05; Figure 1C). Rats that received daily E2 and P4 every other day (dark gray bars) also displayed similar mechanical allodynia to sham animals that received vehicle injections (open bars; *p* > 0.05; Figure 1C).

### A Pharmacological Dose of Progesterone (16 mg/kg) Rapidly Protects Against E2-Elicited Return of CFA-Evoked Mechanical Allodynia in the TMJ

**Figure 2:**
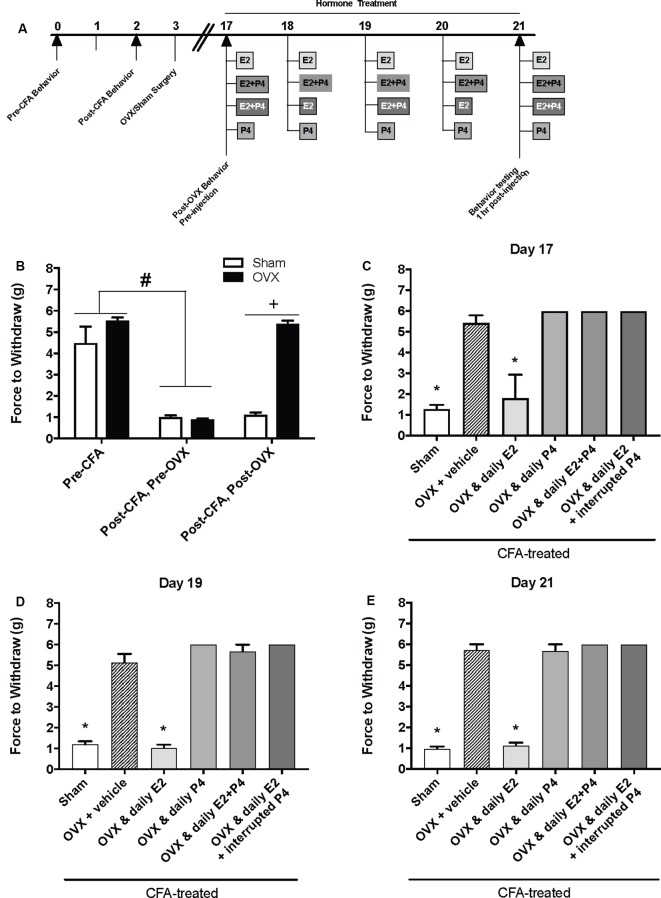
Effects of a pharmacological dose of progesterone (16 mg/kg) on estrogen-exacerbated inflammatory mechanical allodynia in the inflamed rat temporomandibular joint. **(A)** Experimental timeline of behavior testing, ovariectomy, and hormone treatments administered to a different group of rats from Figure 1. Behavior testing was done 24-hrs prior to and following CFA injections followed by either ovariectomy (OVX; closed bars) or sham surgery (open bars). Two-weeks later behavior testing occurred followed by hormone treatments. Hormones were administered every day for 5 days except interrupted progesterone, which was administered on days 17, 19, and 21. Interrupted progesterone administration schedule differs from the interrupted progesterone schedule for rats in Figure 1. Rats in Figure 1 that were in the interrupted progesterone group were tested 25 h after their last treatment of progesterone on day 21. Whereas, rats in Figure 2 that were in the interrupted progesterone group were tested 1 h following administration beginning on day 17. Behavior testing occurred 1 h after hormone injection on days 17, 19, and 21. **(B)** Bar graph showing CFA-evoked mechanical allodynia (pre-CFA vs. post-CFA/pre-OVX) followed by sham (open bars; *n* = 7) vs. OVX (closed bars; *n* = 7) surgery (post-CFA/post-OVX). **(C)** Effects of hormone treatment 1 h after hormone injections on day 17 **(C)**, day 19 **(D)**, and day 21 **(E)** in OVX rats treated over last 5 days with daily vehicle (sesame seed oil; crossed bars; *n* = 7), daily estradiol (50 μg/kg E2; *n* = 6), daily progesterone (16 mg/kg P4; *n* = 6), daily E2 and daily P4 (E2+P4; *n* = 6), or daily E2 with interrupted P4 (*n* = 6) compared to sham treated with daily vehicle (open bars; *n* = 7). ^#^Indicates significant difference from pre-CFA and post-CFA/post-OVX groups. ^+^Indicates significant difference between OVX and sham groups post-surgery. *Indicates significant difference compared to OVX and vehicle control group. Statistical significance was tested at *p* ≤ 0.01.

We then repeated the experiment with an altered timeline to assess whether the observed effects of P4 on mechanical allodynia occurred prior to the last day of hormone treatments. Similar to Figure 1B, we found a significant effect of treatment on mechanical allodynia (*F*_(1,76)_ = 42.8; *p* ≤ 0.05; Figure 2B). CFA again evoked a significant reduction in the force to withdrawal in all rats (*p* ≤ 0.05). In rats that were then ovariectomized, the force to withdraw returned to baseline levels (closed bars; *p* ≤ 0.05) and was significantly greater than the sham surgery rats that remained allodynic (open bars; *p* > 0.05). There was a significant effect of treatment (*F*_(5,32)_ = 282.9; *p* ≤ 0.05), but not time (*F*_(1.9,61.5)_ = 1.931; *p* > 0.05). On day 17 (1st day of hormone treatments), there was a significant effect of treatment 1 h following the first hormone treatment (*F*_(5,32)_ = 83.40; *p* ≤ 0.05; Figure 2C). We found significantly lower mechanical thresholds in ovariectomized females that received daily E2 (light gray bars; *p* ≤ 0.05) and the sham rats that received vehicle treatment (open bars; *p* ≤ 0.05) when compared to all other groups. There was also a significant effect of treatment on day 19 1 h following the 3rd day of hormone injections (*F*_(5,32)_ = 98.61; *p* ≤ 0.05; Figure 2D) with significantly lower mechanical thresholds in ovariectomized females that received daily E2 (light gray bars; *p* ≤ 0.05) and the sham rats that received vehicle treatment (open bars; *p* ≤ 0.05) when compared to all other groups. We did not observe a decrease in mechanical threshold in ovariectomized rats treated with P4 (*p* > 0.05), ovariectomized rats treated with E2 and P4 (*p* > 0.05), or ovariectomized rats treated with E2 and P4 every other day (*p* > 0.05) compared to sham control (open bars) and E2-treated rats (light gray bars). There was also a significant effect of treatment observed 1 h after the last injection on day 21 (after 5 days of hormone treatment paradigm; *F*_(5,32)_ = 161.6; *p* ≤ 0.05; Figure 2E). Sham females that received vehicle injections (open bars) and ovariectomized females that received daily E2 (light gray bars) displayed a significantly lower force to withdraw compared to ovariectomized rats that received vehicle (crossed bars), daily E2 and P4, daily P4 alone, or daily E2 with P4 injected every other day (*p* ≤ 0.05). There was no significant difference between sham controls (open bars) and ovariectomized females treated with E2 (crossed bars; *p* > 0.05). No mechanical allodynia was observed in any group receiving P4 compared to sham or E2 injected rats (*p* > 0.05). On all 3 days, all rats receiving P4 treatment were similar to rats receiving vehicle treatment (crossed bars; *p* > 0.05).

### A 16 μg/kg but Not 16 ng/kg, Dose of Progesterone Protects Against E2-Elicited Return of CFA-Evoked Mechanical Allodynia in the TMJ

**Figure 3:**
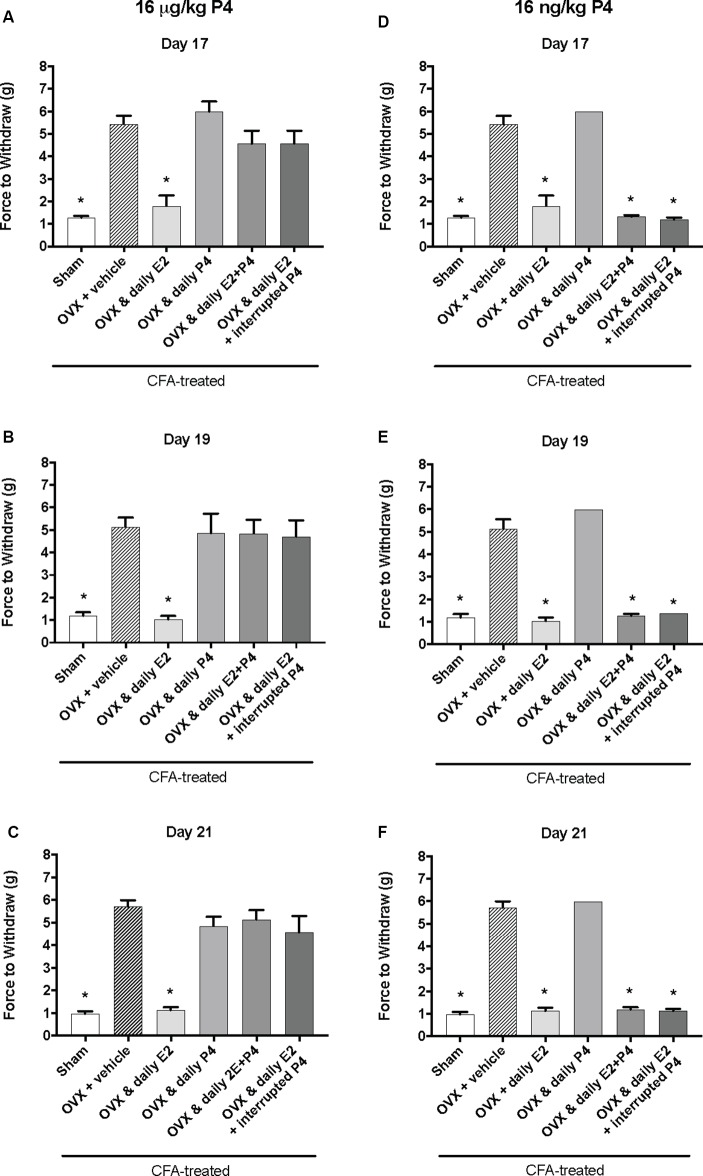
Effects of 16 μg/kg and 16 ng/kg progesterone on estrogen-exacerbated inflammatory mechanical allodynia in the inflamed rat temporomandibular joint. Experimental timeline of behavior testing, ovariectomy, and hormone treatments administered are the same as the timeline in Figure 2. Behavior testing was done 24-hrs prior to and following CFA injections followed by either ovariectomy (OVX) or sham surgery. Two-weeks later behavior testing occurred followed by hormone treatments. Hormones were administered every day over the last 5 days except interrupted progesterone, which was administered on days 17, 19, and 21. Behavior testing was completed on days 17, 19, and 21. Effects of 16 μg/kg of progesterone on mechanical threshold in OVX rats 1 h after last injection of daily vehicle (sesame seed oil; crossed bars; *n* = 6), daily estradiol (E2; *n* = 6), daily progesterone (P4; *n* = 7), daily E2 and daily P4 (E2+P4; *n* = 7), or daily E2 with interrupted P4 (*n* = 7) compared to sham treated with daily vehicle (open bars; *n* = 7) on day 17 **(A)**, day 19 **(B)** and day 21 **(C)**. Effects of 16 ng/ kg of progesterone on mechanical threshold in OVX rats 1 h after last injection of daily vehicle (sesame seed oil; crossed bars; *n* = 7), daily estradiol (E2; *n* = 6), daily progesterone (P4; *n* = 6), daily E2 and daily P4 (E2+P4; *n* = 6), or daily E2 with interrupted P4 (*n* = 6) compared to sham treated with daily vehicle (open bars; *n* = 7) on day 17 **(D)**, day 19 **(E)**, and day 21 **(F)** for 16 ng/kg of progesterone. *Indicates significant difference from OVX and vehicle group. Statistical significance was tested at *p* ≤ 0.01.

We next tested whether a lower dose of P4, 16 μg/kg, could also protect against the E2-elicited return of mechanical allodynia. Using the same treatment paradigm as Figure 2A, we measured mechanical allodynia 1 h after hormone injections on day 17, day 19, and day 21 (days 1, 3, and 5 of hormone treatments). There was a significant effect of treatment (*F*_(5,35)_ = 39.1; *p* ≤ 0.05), but not time (*F*_(1.9,67.9)_ = 0.77; *p* > 0.05). As the treatment effect was again the same on each day, we compared the treatment groups within each day. There was a significant effect of treatment with 16 μg/kg P4 on day 17 (*F*_(5,35)_ = 18.65; *p* ≤ 0.05; Figure 3A), day 19 (*F*_(5,35)_ = 11.76; *p* ≤ 0.05; Figure 3B), and day 21 (*F*_(5,35)_ = 26.13; *p* ≤ 0.05; Figure 3C) of the treatment regimen. On each day, we observed a significantly lower mechanical threshold in the rats receiving daily E2 (light gray bars; *p* ≤ 0.05) and sham controls (open bars; *p* ≤ 0.05) compared to ovariectomized rats receiving vehicle (crossed bars) and all P4 treated rats with or without E2. There was no significant difference in CFA-evoked mechanical allodynia between sham controls (open bars) and ovariectomized rats receiving only E2 (light gray bars) on any treatment day tested (*p* > 0.05) and no mechanical allodynia was observed between any groups receiving P4 (*p* > 0.05).

We then tested whether a sub-physiological dose of P4, 16 ng/kg, would have an effect on E2-elicited return of mechanical allodynia. There was a significant effect of treatment (*F*_(5,32)_ = 315.0; *p* ≤ 0.05), but not time (*F*_(1.9,62.2)_ = 1.3; *p* > 0.05). As the treatment effect was again the same on each day, we compared the treatment groups within each day. There was a significant effect of treatment on day 17 (*F*_(4,27)_ = 162.2; *p* ≤ 0.05; Figure 3D), day 19 (*F*_(4,27)_ = 120.7; *p* ≤ 0.05; Figure 3E), and day 21 (*F*_(4,27)_ = 268.2; *p* ≤ 0.05; Figure 3F). On each testing day, we observed a significantly lower force to withdraw in sham rats (open bars) compared to ovariectomized rats with vehicle treatments (crossed bars; *p* ≤ 0.05) and rats receiving daily P4 only (*p* ≤ 0.05). Significantly lower mechanical thresholds were also observed in ovariectomized rats treated with daily E2 (light gray bars) or in combination with daily or interrupted 16 ng/kg P4 compared to ovariectomized rats with vehicle treatments (crossed bars; *p* ≤ 0.05) and compared to ovariectomized rats with only P4 treatment (*p* ≤ 0.05).

### Plasma Progesterone Levels in Female Rats That Received 16 mg/kg or 16 μg/kg, but Not 16 ng/kg, of Progesterone Were Significantly Higher Than Ovariectomized Females

Plasma P4 levels were measured following high E2 + P4 (16 mg/kg, 16 μg/kg, or 16 ng/kg) and in OVX females receiving the vehicle injection (sesame seed oil). The pharmacological 16 mg/kg dose of P4 resulted in plasma P4 levels significantly higher than OVX control females (40.02 ± 2.08 (37.11–48.04) ng/ml vs. 0.82 ± 0.16 (0.45–1.4) ng/ml; Mann–Whitney *U* = 0, *n*_1_ = 5, *n*_2_ = 5, *p* ≤ 0.05), females that received 16 μg/kg P4 (2.25 ± 0.40 (1.22–3.26) ng/ml; Mann–Whitney *U* = 0, *n*_1_ = 5, *n*_2_ = 5, *p* ≤ 0.05), and from females that received 16 ng/kg P4 (mean = 1.96 ± 0.41 (0.41–2.67) ng/ml; Mann–Whitney *U* = 0, *n*_1_ = 5, *n*_2_ = 5, *p* ≤ 0.05; data not shown). Also, plasma P4 levels following the 16 μg/kg dose were significantly different from ovariectomized control females (Mann–Whitney *U* = 2, *n*_1_ = 5, *n*_2_ = 5, *p* ≤ 0.05), but did not differ from plasma P4 levels following the 16 ng/kg dose (Mann–Whitney *U* = 9, *n*_1_ = 5, *n*_2_ = 5, *p* > 0.05). Plasma P4 levels of females that received 16 ng/kg P4 did not differ from ovariectomized control females (Mann–Whitney *U* = 4, *n*_1_ = 5, *n*_2_ = 5, *p* > 0.05).

### Allopregnanolone (0.16 mg/kg) Rapidly, but Only Acutely, Protects Against E2-Elicited Return of CFA-Evoked Mechanical Allodynia in the TMJ

**Figure 4:**
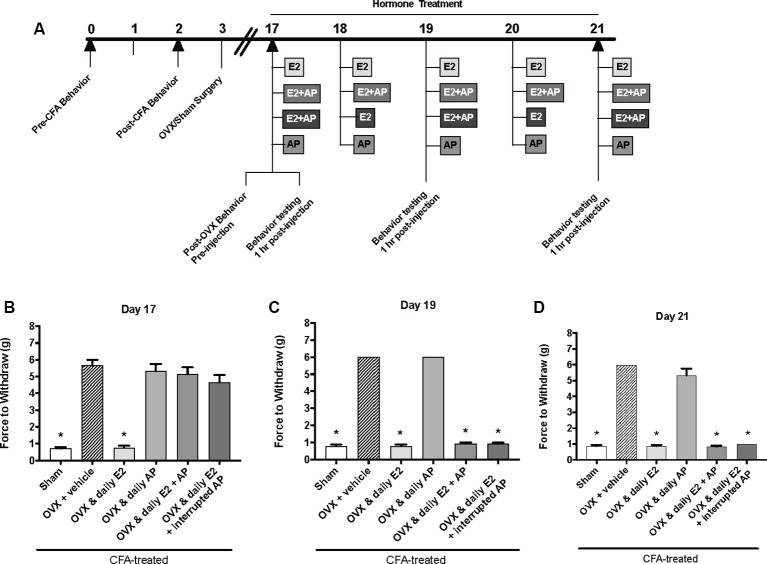
Effects of 0.16 mg/kg allopregnanolone on estrogen-exacerbated inflammatory mechanical allodynia in the inflamed rat temporomandibular joint. **(A)** Experimental timeline of behavior testing, ovariectomy, and hormone treatments administered. Behavior testing was done 24-hrs prior to and following CFA injections followed by either ovariectomy (OVX) or sham surgery. Two-weeks later behavior testing occurred followed by hormone treatments. Hormones were administered every day over the last 5 days except interrupted allopregnanolone, which was administered on days 17, 19, and 21. Behavior testing was completed on days 17, 19, and 21. Bar graphs showing mechanical threshold in OVX rats on day 1 **(B)**, day 3 **(C)**, and day 5 **(D)** 1 h after last injection of daily vehicle (sesame seed oil; crossed bars; *n* = 6), daily estradiol (E2; *n* = 6), daily allopregnanolone (AP; *n* = 6), daily E2 and daily AP (E2+AP; *n* = 7), or daily E2 with interrupted AP (*n* = 6) compared to sham treated with daily vehicle (open bars; *n* = 6). *Indicates significant difference from OVX and vehicle group. Statistical significance was tested at *p* ≤ 0.01.

The same behavior testing paradigm was used here, but AP was injected instead of P4 and mechanical allodynia was detected on day 17, day 19, and day 21 (days 1, 3, and 5 of hormone treatments; Figure 4A). We found a significant interaction between treatment groups across time (*F*_(10,62)_ = 39.1; *p* ≤ 0.05). There was a significant effect of treatment (*F*_(5,31)_ = 295.6; *p* ≤ 0.01) and a significant effect of time (*F*_(2,62)_ = 66.2; *p* ≤ 0.05). When comparing treatment groups within each day, there was a significant effect of treatment on the first day of hormone treatments (day 17; *F*_(5,31)_ = 47.54; *p* ≤ 0.05; Figure 4B) 1 h after hormone injections. The force to withdraw was significantly lower in ovariectomized rats treated daily with E2 (light gray bars; *p* ≤ 0.05) and the sham controls (open bars; *p* ≤ 0.05) when compared to ovariectomized females receiving vehicle (crossed bars), daily AP, daily E2 and AP, or daily E2 and AP every other day. No mechanical allodynia was observed in any group receiving AP compared to sham (open bars) or E2 injected rats (light gray bars; *p* > 0.05). There was also a significant effect of treatment on day 19 (*F*_(5,31)_ = 1,762; *p* ≤ 0.05) and day 21 (*F*_(5,31)_ = 191.7; *p* ≤ 0.05). However, on day 19 (Figure 4C) and day 21 (Figure 4D), mechanical threshold was significantly lower in all ovariectomized females receiving daily E2 with or without AP compared to receiving only AP (*p* ≤ 0.05). Similar to day 17, rats receiving daily AP did not display allodynia on day 19 or day 21 and were similar to vehicle treated rats (*p* > 0.05).

## Discussion

TMD pain is a hallmark pain disorder more prevalent in women that is greatest during the reproductive years, dissipates after menopause (LeResche et al., 2003), and can reemerge with estrogen replacement therapy (LeResche, 1997; Wise et al., 2000). Progesterone and it’s metabolite allopregnanolone have anti-inflammatory and antinociceptive properties (for review, see Coronel et al., 2016a), while estrogen appears to be pronociceptive at high pharmacological doses (Wu et al., 2010; Kou et al., 2011; Ralya and McCarson, 2014; Pratap et al., 2015). Here, we hypothesized that ovariectomy would attenuate inflammatory allodynia in the rat TMJ and that both progesterone and allopregnanolone would attenuate the pharmacological estrogen-evoked reemergence of inflammatory TMJ allodynia in female rats. Overall, we report that: (1) ovariectomy attenuated CFA-evoked mechanical allodynia at the TMJ; (2) pharmacological estrogen treatment triggered the reemergence of mechanical allodynia which was attenuated by co-treatment with progesterone at 16 mg/kg and 16 μg/kg, but not 16 ng/kg; and (3) the progesterone metabolite allopregnanolone was also able to attenuate E2-evoked reemergence of allodynia, but only on the first injection day.

CFA injection at the TMJ triggered significant mechanical allodynia as measured by von Frey filaments, similar to previous studies (Ren, 1999; Guo et al., 2010; Villa et al., 2010). A limitation of this method is that deep pain in the joint is not detected, however, our data and previous studies indicate that mechanical allodynia can be readily detected at the cutaneous tissues surrounding the joint. Thus, while CFA-injection at the TMJ provides an inflammatory pain model, it may not fully capture the TMJ damage and resulting deep pain in the joint observed in the clinic. The repeated open-mouth procedure to induce TMJ dysfunction and orofacial mechanical allodynia (Wang et al., 2018) may provide a more clinically-relevant model to utilize to examine the potential mechanisms underlying the role of estrogen and progesterone on TMD pain. This model, however, does not produce persistent pain required for our experimental design. Future studies could integrate CFA- or carrageenan-evoked joint inflammation into the open-mouth procedure to create more persistent TMD-like pain conditions.

In order to observe the effects of ovariectomy on inflammatory TMD pain, CFA was injected prior to ovariectomy. In this paradigm, ovariectomy resulted in antinociception when compared to the sham controls. However, in a different paradigm where CFA was injected after ovariectomy, the ovariectomized rats experienced nociception, as measured by meal duration (Kramer and Bellinger, 2009). Data from these two different paradigms suggest timing of ovariectomy in relation to CFA injection, may affect the development of nociception. However, further studies are warranted to determine if ovariectomy before injury affects the development of nociceptive behaviors or the response to hormonal treatment. Following ovariectomy, mechanical sensitivity subsided to basal levels while sham animals remained allodynic, in concurrence with previous findings (Wu et al., 2010). While mechanical sensitivity decreased, the rats from that study did not return to basal levels of sensitivity, which is likely due to a difference in unilateral vs. bilateral CFA injections. Interestingly, when our rats were treated with pharmacological estrogen a reemergence of mechanical allodynia was observed, supporting a pronociceptive role of high doses of estrogen in the TMJ, and similar to previous reports (Kou et al., 2011; Zhang et al., 2012a; Wang et al., 2013; Fejes-Szabó et al., 2018). In opposition, estrogen treatment can also attenuate CFA-induced TMJ nociception (Kramer and Bellinger, 2009). The opposing reports on estrogen’s pronociceptive effects are likely due to differences in estrogen dose, timing of injections, and model used between studies. A low, continuously administered physiological dose of E2 (750 ng/6 μl per day by a pump and an injection of 2.5 μg E2 every 5 days) administered prior to and during TMJ inflammation appears to be antinociceptive on orofacial pain (Kramer and Bellinger, 2009). We report that a higher pharmacological E2 dose (50 μg/kg) administered after TMJ inflammation is pronociceptive. Together these data are interesting because they point to a possibility that consistent, physiological or low E2 is protective against orofacial pain when administered prior to and during TMJ inflammation, but large fluctuations in or high doses of E2 administered after TMJ inflammation may enhance orofacial pain. This is supported by clinical reports that migraine is worsened during the late luteal and follicular phases, and during menstruation (MacGregor et al., 2010; Gupta et al., 2011; Shuster et al., 2011). Further, our data supports the clinical reports of women experiencing TMD pain reemergence while undergoing estrogen-replacement therapy. On the other hand, the same pharmacological dose of E2 that was used in our study was also reported to be antinociceptive in the formalin-inflamed rat TMJ model (Fischer et al., 2008). Estrogen may modulate formalin-induced inflammation differently than CFA-induced inflammation, resulting in either a pronociceptive or antinociceptive effect.

When estrogen was administered daily in the presence of progesterone, the development of allodynia was not observed. Others have also found that progesterone reduces the development of persistent pain in animal models of inflammatory pain (Ren et al., 2000), diabetic neuropathy (Leonelli et al., 2007), and nerve injury (Roglio et al., 2008, 2009; Coronel et al., 2011; Kim et al., 2012). Our data considered in the context of these studies indicate that progesterone may counter estrogen-evoked nociception. In support, a previous study reported that estrogen-evoked hyperalgesia was diminished when estrogen treatment was combined with progesterone (Ji et al., 2005). Our findings support this protective effect of progesterone on estrogen-evoked pain and further we report that this occurs in the trigeminal system in a rat model of inflammatory pain at the TMJ. Interestingly, we also found that the effects of both estrogen and progesterone occurred quickly. Evidence of estrogen-evoked reemergence of mechanical allodynia at the TMJ was observed on the first day of hormone treatments 1 h following injection. When progesterone was administered on an interrupted schedule instead of daily with estrogen, attenuation of mechanical allodynia occurred within 1 h of injections and as early as the first day on the 5-day treatment schedule (day 17 on the overall timeline). These data indicate that progesterone has quick-acting, but not long-lasting, actions on pain relief in the TMJ.

The optimal pharmacological dose of progesterone used in different animal models, such as traumatic brain or spinal cord injury and neuropathic pain, is 16 mg/kg (Pettus et al., 2005; Labombarda et al., 2011; Coronel et al., 2014, 2017). This dose reduces edema, improves cognitive function, and prevents neuronal loss (Cutler et al., 2005; Pettus et al., 2005; Kasturi and Stein, 2009; Labombarda et al., 2009; Jarahi et al., 2014). While, we found a protective effect of 16 mg/kg on pain, we also tested two physiological to sub-physiological doses of progesterone (16 μg/kg or 16 ng/kg) and observed rapid attenuation with the 16 μg/kg dose of progesterone but not with 16 ng/kg dose. In support, others have also reported that timing, dosage, and duration of progesterone treatment is vital for attenuating nociceptive behaviors (Verdi et al., 2013; Jarahi et al., 2014; Liu et al., 2014). Progesterone has genomic effects at the intracellular progesterone receptor but also has rapid, non-genomic effects at membrane progesterone receptors (mPRα, mPRβ, mPRδ, mPRε) and sigma receptors (Johannessen et al., 2011). Progesterone’s binding affinity (in ascending order) is 2.7 nM for mPRδ, 2.9 nM for mPRε (Pang et al., 2013), 3.4 nM for iPR (Ogle, 1980), 7 nM for mPRα, 12 nM for mPRβ (Hanna et al., 2006), 239 nM for sigma 1 receptor (S1R)s, and 441 nM for sigma 2 receptors (Johannessen et al., 2011). Given the rapid effects of progesterone in the present study, progesterone may be acting at mPRs rather than iPRs. In the present study, 16 mg/kg progesterone produced ~41.75 ng/ml (or 127.26 nM) in the plasma and thus has the potential to bind to and activate all of the progesterone receptors to attenuate mechanical allodynia in the current study. Plasma progesterone levels following 16 μg/kg and 16 ng/kg were 2.25 ng/ml (7.16 nM) and 1.96 ng/ml (6.23 nM), respectively. Thus, the lower dose could activate mPRδ and mPRε, while 16 μg/kg dose would also activate mPRα. Both mPRδ and mPRε are stimulatory G-protein coupled receptors (GPCR) that increase in cAMP production (Hanna et al., 2006), which is associated with an increase in nociception (for review see Skyba et al., 2004). We elucidate that 16 ng/kg progesterone dose acting at mPRδ and mPRε would have pronociceptive properties *via* increased cAMP production, which could explain why this dose did not protect against the return of mechanical allodynia. On the other hand, 16 μg/kg of progesterone would also activate mPRα receptor, which is a GPCR that activates inhibitory G proteins, thus inhibiting cAMP production (Zhu et al., 2003). Activation of mPRα could inhibit cAMP production, thereby, attenuating mechanical allodynia, as observed in our study. Taken together, our data indicate an important role for the mPRs in our TMD pain model, however, testing this hypothesis is currently limited as there are no commercially available antagonists for these receptors.

Another possible explanation for the antinociceptive effect of 16 μg/kg, but not 16 ng/kg, progesterone (despite similar plasma progesterone levels), could be linked to differences in the serum progesterone to estrogen ratio. Unfortunately, there appear to be no studies on the effect of altered progesterone to estrogen ratios in animal pain models for female-prevalent disorders linked to gonadal hormones. In the present study, all hormone-treated groups received the same pharmacological dose of estradiol (50 μg/kg) and while the plasma progesterone levels did not differ between the two lowest doses of progesterone, the resulting plasma progesterone levels from the 16 μg/kg, but not 16 ng/kg, were significantly greater than ovariectomized rats. We did not measure plasma estradiol levels, but this possibility warrants future studies to detect a potential role of ovarian hormonal ratios in nociceptive orofacial behavior.

The acute effects of these two major gonadal hormones may be due to opposing activities on both pain and inflammation. Estrogen enhances nociception by upregulating inflammatory mediators (Kou et al., 2011; Pratap et al., 2015), upregulating injury-induced inflammatory processes (Flake et al., 2006), modulating ion channel expression (Wu et al., 2010, 2015; Hu et al., 2012; Bi et al., 2017), and increasing sensory neuron excitability in rats (Flake et al., 2005). Progesterone, on the other hand, decreases nociception by attenuating inflammatory microglial activation (Labombarda et al., 2011; Garay et al., 2012), inhibiting injury-induced upregulation of proinflammatory mediators (Garay et al., 2012; Coronel et al., 2014, 2016b; Grandi et al., 2016), and inhibiting ion channel activity (Johannessen et al., 2011; Kelley and Mermelstein, 2011). Interestingly, progesterone, through actions at the intracellular progesterone receptor, inhibits the ability of estrogen to modulate gene expression and thus may underlie the attenuation of estrogen’s effects on inflammatory mediators (Kraus et al., 1995). So, it is possible in the present study that progesterone is counteracting estrogen’s pronociceptive effects at the TMJ *via* inhibiting inflammatory mediators and preventing the estrogen-evoked upregulation of ion channel expression that triggers hypersensitivity. It is unclear where the site of action of gonadal hormones are in this system. Progesterone receptors are expressed throughout regions of the brain, such as thalamus, spinal cord, and medulla (Pang et al., 2013), as well as the trigeminal ganglia (Manteniotis et al., 2013). Possibilities include direct action at trigeminal sensory neurons or activity at the trigeminal nucleus caudalis where primary afferents enter the central nervous system. Proestrus estrogen levels upregulate neurotransmitter receptors and pro-inflammatory cytokines within the trigeminal ganglia, as well as, the superficial laminae of the upper cervical cord region (Vc/C_1-2_; Puri et al., 2011) and increase neural activity and excitability in the trigeminal nucleus caudalis of the medullary spinal cord (Okamoto et al., 2003, 2008; Bereiter et al., 2005). Future studies are warranted to examine the effects of the hormone treatments utilized in the current study on the neural activity in the trigeminal nucleus caudalis.

Alternatively, progesterone’s acute effects on pain may involve the S1R. The S1R is a non-opioid chaperone receptor located in the plasm membrane of the endoplasmic reticulum (Hayashi and Su, 2003) expressed in regions associated with pain regulation, such as dorsal root ganglia, spinal cord, thalamus, and rostroventral medulla of female mice (Sánchez-Fernández et al., 2014) and the spinal cord, thalamus, and sciatic of male rats (Alonso et al., 2000). S1R is reported in the trigeminal ganglia of male mice (Yoon et al., 2015), but expression in female trigeminal sensory neurons currently unknown. Activation of S1R elicits nociceptive responses, which can be reversed with SR1 antagonists (Kim et al., 2008; Gris et al., 2014; Parenti et al., 2014; Pyun et al., 2014; Roh and Yoon, 2014; Tejada et al., 2014; Entrena et al., 2016) or in S1R knockout mice (Entrena et al., 2009; de la Puente et al., 2009). Progesterone is a potent S1R antagonist (Johannessen et al., 2011; Zamanillo et al., 2013) and can inhibit nociception (Maurice et al., 2001; Ueda et al., 2001; Maurice and Su, 2009). In the current study, it is possible that high levels of progesterone could be acting at the S1R to counteract estrogen’s effects on inflammatory orofacial allodynia.

The rapid attenuation by progesterone may also involve its metabolite, allopregnanolone. Allopregnanolone synthesis from progesterone involves two enzymes, 5α-reductase and 3α-hydroxysteroid oxidoreductase. The former converts progesterone to 5α-dihydroprogesterone (5α-DHP), while the latter converts 5α-DHP to allopregnanolone (Schumacher et al., 2014). Allopregnanolone has been shown to have antinociceptive effects. Allopregnanolone attenuates diabetes-induced neuropathy (Afrazi and Esmaeili-Mahani, 2014), postoperative pain (Fujita et al., 2018), inflammatory pain (Charlet et al., 2008; Ocvirk et al., 2008), sciatic nerve ligation nociception (Meyer et al., 2008), and chemotherapy-induced nociception (Meyer et al., 2011). In the present study, we observed a rapid attenuation in the reemergence of mechanical allodynia within 1 h of injection on day 1. This is in agreement with previous studies that investigated the effects of allopregnanolone in animal models of neuropathic pain (Charlet et al., 2008; Meyer et al., 2008, 2011; Ocvirk et al., 2008; Kawano et al., 2011; Svensson et al., 2013; Afrazi and Esmaeili-Mahani, 2014; Fujita et al., 2018).

Surprisingly, allopregnanolone treatment did not attenuate the return of mechanical allodynia on day 3 or 5 of hormone treatment. This is not in agreement with a study that reported allopregnanolone suppressed diabetes-induced thermal hyperalgesia up to 7 weeks (Afrazi and Esmaeili-Mahani, 2014). The variance could be due to different dosage effects. Their study utilized high doses of allopregnanolone (5 mg/kg or 20 mg/kg) compared to our dose of 0.16 mg/kg. Perhaps a high dose of allopregnanolone is necessary to provide continuous attenuation of the return of orofacial mechanical allodynia after several days of repeated hormone treatment. It was previously reported that the most potent dose of allopregnanolone in reducing nociception is 0.16 mg/kg, but effects over time were not investigated (Ocvirk et al., 2008). It could be that allopregnanolone underlies protective effects on pain early on in treatment, but that progesterone’s continual attenuation of the return of orofacial mechanical allodynia on the other days may involve a mechanism such as the sigma1 receptor. However, future studies are warranted to determine the reason for the observed effects of allopregnanolone in the current study on the reemergence of mechanical allodynia following estrogen treatment.

Allopregnanolone may have acute antinociceptive effects through the GABA_A_ receptor. This is supported by reports that allopregnanolone decreases pain in neuropathy models (Afrazi and Esmaeili-Mahani, 2014; Huang et al., 2016). This effect may then be attenuated on day 3 and day 5 due to the development of tolerance to allopregnanolone. Repeated daily administration of allopregnanolone was reported to induce tolerance to allopregnanolone’s anticonvulsant (Członkowska et al., 2001) and hypothermic properties (Palmer et al., 2002). Also a 90-min exposure to allopregnanolone triggers an increase in allopregnanolone in brain regions important in tolerance development (Zhu et al., 2004; for review see Türkmen et al., 2011). Interestingly, prolonged exposure to allopregnanolone alters GABA_A_ receptor subunits, resulting in a decrease in sensitivity to allopregnanolone (Zhu et al., 2004; Türkmen et al., 2006, 2008). Alternatively, on days 3 and 5 estrogen may be further increasing the excitability of TMJ neurons (Flake et al., 2005), increasing inflammatory mediators, and increasing GABA_A_ receptor in the trigeminal ganglia (Puri et al., 2011); all of which could be opposing protective effects of allopregnanolone. Based on these lines of evidence, studies examining potential effects of progesterone and allopregnanolone on the GABA_A_ receptor in the trigeminal ganglia are needed to understand the mechanism underlying the protective role of progesterone in our treatment paradigm.

Overall, we show that removal of the endogenous source of ovarian hormones after orofacial inflammation relieves mechanical allodynia in the TMJ of female rats. We also showed rapid attenuation of the high estrogen-evoked return of mechanical allodynia by two different doses of progesterone and acute, rapid attenuation by allopregnanolone. These data suggest the allopregnanolone may provide short-term relief, whereas, progesterone may provide continual relief in the reemergence of TMD pain in post-menopausal women undergoing estrogen replacement therapy.

## Data Availability Statement

All datasets generated for this study are included in the article.

## Ethics Statement

All studies were approved by Texas Woman’s University Institutional Animal Care and Use Committee. Experiments conformed to federal guidelines and the committee for Research and Ethical Issues of the International Association for the Study of Pain.

## Author Contributions

RH contributed to experimental design, conducting experiments, data analysis and interpretation, and preparation of the manuscript. WB contributed to experimental design, conducting experiments, data analysis and interpretation, and approval of the manuscript. ST contributed to conducting experiments, data analysis and interpretation, and approval of the manuscript. LU and PK contributed to experimental design, data analysis and interpretation, and editing the manuscript. DA contributed to experimental design, data analysis and interpretation, and preparation of the manuscript.

## Conflict of Interest

The authors declare that the research was conducted in the absence of any commercial or financial relationships that could be construed as a potential conflict of interest.
